# AI-Driven Heart Failure Decision Support in Skilled Nursing Facilities

**DOI:** 10.1016/j.jaccas.2026.108561

**Published:** 2026-06-03

**Authors:** Emma Glajchen, Mills Reed, Takeshi Uemura, Johanna Contreras, Zoe Ozols, Annapoorna S. Kini, Jeffrey Bander

**Affiliations:** a Icahn School of Medicine at Mount Sinai, New York, New York, USA; b Division of Cardiology, Mount Sinai Fuster Heart Hospital, Mount Sinai West, New York, New York, USA; c ProviderLoop, New York, New York, USA

**Keywords:** acute heart failure, angiotensin receptor blockers, chronic heart failure, diastolic heart failure, diuretic, echocardiography, hemodynamics, orthopnea, preserved ejection fraction, systolic heart failure

## Abstract

Heart failure management in skilled nursing facilities (SNFs) is complicated by limited access to specialists, incomplete clinical documentation, and patients with complex comorbidities. Artificial intelligence clinical decision support systems have been developed mostly for acute hospital settings but not for SNF settings. We present ForeSight-HF, an artificial intelligence clinical decision support tool designed specifically for SNF heart failure management. Built on a large language model with HIPAA-compliant infrastructure, ForeSight-HF synthesizes unstructured clinical documentation to generate heart failure likelihood scores, exacerbation risk scores, and individualized management recommendations. We illustrate its utility through 2 contrasting cases: a Warm/Wet (congested, well-perfused) patient for whom the system recommended intensified diuresis, and a Cold/Dry (hypovolemic, hypoperfused) patient for whom it recommended diuretic de-escalation to prevent harm. ForeSight-HF may help close a persistent care gap in postacute heart failure management.

Heart failure (HF) affects more than 64 million people worldwide, and nearly 25% of Medicare fee-for-service beneficiaries hospitalized for HF are readmitted within 30 days.^1^^,^^2^ One in 5 Medicare beneficiaries discharged from the hospital receives postacute care in a skilled nursing facility (SNF) at a cost exceeding $28 billion annually.^3^ Despite the clinical complexity of this population, SNFs often face limited access to specialists, variable documentation quality, and resource constraints that complicate guideline-directed HF management.^4^ In this setting, patients are simultaneously vulnerable to undertreatment and overtreatment; excessive diuresis in particular can precipitate hypotension and acute renal dysfunction.

**Take-Home Messages**

• SNFs represent a persistent gap in heart failure care, where patients are simultaneously vulnerable to undertreatment and overtreatment.
• AI clinical decision support systems designed specifically for the SNF setting can provide valuable clinical decision and triage support; ForeSight-HF is, to our knowledge, the first such system developed for this environment.

Artificial intelligence (AI) has shown growing promise in HF diagnosis and clinical decision making, and the American Heart Association's 2024 Scientific Statement recognizes its potential to improve cardiovascular outcomes.^5^ However, existing artificial intelligence clinical decision support systems (AI-CDSSs) have been developed in hospital settings; to our knowledge, no AI-CDSS has been designed for SNFs.^6^ Here, we present ForeSight-HF, an AI-CDSS designed for SNF HF management, through 2 case examples representing contrasting Forrester hemodynamic quadrants.^7^

## The ForeSight-HF System

ForeSight-HF uses OpenAI's GPT-5 mini model (accessed late 2025) via HIPAA-compliant infrastructure to generate real-time risk assessments, hemodynamic classification, and management recommendations. Clinical specificity is achieved through prompt engineering that layers disease-specific instructions onto the model's foundational medical knowledge. The 400,000-token context window enables processing of extensive clinical documentation without truncation, supporting HF triage even when documentation is sparse.

### System architecture

ForeSight-HF employs a 2-stage agentic architecture. In stage 1, a note-processing bot ingests unstructured clinical documentation, extracting information from the medical record at admission to the SNF, to establish a baseline; in stage 2, a bot runs weekly reviews to incorporate new information. The decision-making module then compares baseline and updated data to generate clinical assessments and management recommendations.

### Scoring system

ForeSight-HF generates 3 assessments. The *Heart Failure Likelihood Score* is a 20-point scale incorporating 8 clinical domains: echocardiogram data, N-terminal pro–B-type natriuretic peptide (NT-proBNP) and/or B-type natriuretic peptide (BNP) values, documented HF diagnosis, current medications, cardiac devices, comorbidities, symptoms, and temporal trends. The *Confidence Score* (0-1.0) reflects diagnostic certainty. The *Exacerbation Risk Score*, categorized as low (0-4), moderate (5-6), or high (≥7), incorporates acute weight changes, NT-proBNP and/or BNP trends, creatinine changes, symptoms, vital signs, and hemodynamic classification.

### Forrester hemodynamic classification

The system assigns continuous scores from −1 to +1 for volume status (Wet/Dry) and perfusion status (Cold/Warm), weighting vital sign trends, natriuretic peptide and weight changes, and physical examination findings. This continuous approach captures the spectrum of hemodynamic presentations more precisely than discrete quadrant assignment (Table 1).

**Table 1:** Forrester Hemodynamic Quadrants

Quadrant	Hemodynamic Profile
Warm/Dry	Euvolemic, well-perfused
Warm/Wet	Congested, well-perfused
Cold/Dry	Hypovolemic, hypoperfused
Cold/Wet	Congested, hypoperfused

#### Intended use

ForeSight-HF is designed to supplement physician judgment. Assessments are reviewed by physicians during weekly interdisciplinary team meetings on the ProviderLoop platform, during which a remote cardiologist discusses AI-generated findings with on-site clinical staff. Nursing staff from the SNF may receive notifications prompting escalation to physician review, but all clinical decisions require physician authorization. In this proof-of-concept phase, system outputs were used for illustrative purposes only and did not guide clinical care.

## Clinical Cases

The following cases represent contrasting Forrester hemodynamic quadrants (Warm/Wet and Cold/Dry). Clinical presentations are derived from AI-generated summaries of each patient's clinical chart.

### Case 1: guiding aggressive diuresis for acute decompensation (Warm/Wet)

#### Clinical presentation

A 74-year-old man with HF with reduced ejection fraction (EF) (EF 10%), atrial fibrillation on anticoagulation, and history of pulmonary embolism was receiving SNF care. His guideline-directed medical therapy (GDMT)^8^ included metoprolol succinate 25 mg daily, mineralocorticoid antagonist (MRA) 25 mg daily, dapagliflozin 10 mg daily, and torsemide, increased from 20 mg to 40 mg daily for congestion. Over 4 weeks, serial weights demonstrated progressive gain from 156 to 169 lb. During 1 week, the gain exceeded 5 lb and was documented as “clinically significant.” Examination revealed 2+ bilateral pedal edema and orthopnea. BNP values remained low (39-102 pg/mL) despite clear volume overload. Blood pressures were stable (107/68-142/78 mm Hg) without hypotension.

#### ForeSight-HF assessment

HF Likelihood Score 10.5/20, derived from echocardiogram data showing severely reduced EF of 10% (4 points), diagnosis of chronic systolic HF (2 points), current medications including spironolactone and dapagliflozin (2 points), symptoms of orthopnea and lower extremity edema (2 points), and comorbidity of atrial fibrillation (0.5 points). Laboratory values contributed 0 points because the chart did not include NT-proBNP values and the most recent BNP was treated as no significant change. congestive heart failure Determination: Present (HF with reduced EF); Confidence Score: 0.75 (moderate-to-high confidence based on severely reduced EF and use of HF-specific medications).

##### Forrester classification

For volume status (Wet/Dry), the system weighted progressive weight gain exceeding 5 lb per week, 2+ bilateral pedal edema, and orthopnea against low BNP values. The system integrated the clinical findings to identify volume overload, yielding a Wet/Dry Score of −0.8. For perfusion status (Cold/Warm), blood pressures consistently above hypotension thresholds and stable heart rates without bradycardia, combined with absence of cool extremities or signs of shock, supported adequate tissue perfusion (Cold/Warm +1.0). The patient was classified as Warm/Wet.

#### AI-generated management recommendations

The Warm/Wet classification was flagged as high exacerbation risk, triggering the volume overload management pathway. Given recent torsemide escalation, the system recommended maintaining current diuretic therapy with intensified monitoring (daily weights, vital signs every 6-8 hours, daily basic metabolic panel for 3-5 days). If weight remained unchanged or increased after 48 to 72 hours with stable renal function, metolazone 2.5 mg daily was recommended for sequential nephron blockade with repeat laboratory assessment 24 hours after initiation. Safety parameters specified diuretic reduction if creatinine rose above 1.5 mg/dL.

#### Intervention and outcome

The patient remained at the SNF for the following 4 months, managed independently of the AI system. Management continued with torsemide 40 mg daily and intensified monitoring. Despite this, weight continued to increase from 167 to 180 lb over subsequent weeks and bilateral lower extremity edema persisted. Cardiology was consulted and sacubitril/valsartan 24 to 26 mg twice daily was initiated. Following initiation, weight stabilized, edema resolved, and repeat echocardiogram demonstrated EF improvement from 10% to 45%. Creatinine rose slightly from 0.94 to 1.49 mg/dL, warranting continued monitoring but stabilized. The patient remained in the SNF without need for hospitalization or escalation of care throughout this period.

#### Clinical significance

This case demonstrates the role of ForeSight-HF as a triage tool for subacute decompensation manageable within the SNF setting. The system correctly identified volume overload despite paradoxically low BNP values, synthesizing multiple clinical inputs rather than deferring to biomarkers alone—a valuable capability in SNFs where laboratory access is often limited. The patient's subsequent recovery and EF improvement to 45% after GDMT intensification illustrate the potential benefit of early GDMT optimization.

### Case 2: recognizing overdiuresis and recommending therapy de-escalation (Cold/Dry)

#### Clinical presentation

An 82-year-old woman with HF with preserved EF (EF 56%), hypertension, chronic kidney disease stage 3b, diabetes, and dementia was in long-term SNF care. Her regimen included torsemide 10 mg daily, empagliflozin 10 mg, but not MRA. Serial BNP values were consistently low (40-61 pg/mL). Weight had gradually increased from the mid-150s lb to more than 165 lb over a 3-month follow-up period, but nutrition notes documented improved oral intake, suggesting the weight gain was at least partially nutritional rather than fluid-related. Creatinine had risen from 1.2 to 1.73 mg/dL, and multiple hypotensive episodes were recorded (nadir 94/53 mm Hg). Physical examination showed no pulmonary congestion, jugular venous distension, or peripheral edema.

##### ForeSight-HF assessment

HF Likelihood Score: 5/20—documented HF diagnosis (2 points), torsemide use (1 point), chronic kidney disease and diabetes comorbidities (1 point), dyspnea (1 point). Preserved EF and low BNP contributed no points. The primary data gap was absence of diastolic function assessment to evaluate for HF with preserved EF. CHF Determination: Present (unspecified phenotype pending diastolic assessment). Confidence Score: 0.6.

##### Forrester classification

For volume status (Wet/Dry), BNP remained persistently low over 3 months (40-61 pg/mL, well below the system's >400 pg/mL threshold for patients older than 75). There was no acute weight gain (>5 lb/wk) typical of fluid overload, and examination showed no pulmonary congestion, rales, orthopnea, or jugular venous distension. The gradual weight increase was attributed to improved oral intake per nutrition notes, and rising creatinine on ongoing diuretic therapy suggested overdiuresis rather than euvolemia (Wet/Dry +0.5, dry). For perfusion status (Cold/Warm), stable heart rates (64-73 beats/min) and absence of cool extremities, cyanosis, or overt shock were reassuring, but multiple blood pressure readings below hypotension thresholds (94/53, 101/56, 102/52 mm Hg) indicated mildly impaired perfusion (Cold/Warm −0.3). The patient was classified as Cold/Dry, indicating overdiuresis.

##### AI-generated management recommendations

The Cold/Dry classification was determined as low exacerbation risk, triggering therapy de-escalation. The system recommended reducing torsemide from 10 mg to 5 mg daily given persistent hypotension, with blood pressure monitoring every 6 hours and renal function reassessment within 48 hours. Given the low blood pressure, the system advised against adding renin-angiotensin-aldosterone system inhibitors or MRAs. The reduced regimen was to be maintained if renal function and blood pressure stabilized, with cautious diuretic increase only if early volume overload appeared (weight gain >2-3 lb/24 hours, rising BNP).

#### Intervention and outcome

The patient remained at the SNF for 4 months, managed independently of the AI system, continuing torsemide 10 mg, starting losartan 25 mg, and spironolactone 25 mg. Clinical status stabilized: weight remained between 164 and 169 lb without edema, BNP remained low (50-58 pg/mL), and creatinine improved from 1.73 to a nadir of 1.39 mg/dL. Blood pressure varied across visits, trending downward from 150/71 mm Hg to a low of 90/53 mm Hg over the 4-month period, requiring ongoing clinical balancing of fall risk associated with orthostatic hypotension against adequate HF and hypertension control. The patient did not require hospitalization or escalation of care during this period. Intercurrent conditions including urinary tract infection and influenza were addressed within the context of ongoing long-term SNF care.

#### Clinical significance

This case illustrates the potential of ForeSight-HF to prevent iatrogenic harm. The system's integration of BNP trends, weight patterns, and clinical context to distinguish nutritional weight gain from fluid overload exemplifies the nuanced reasoning required for safe HF management in older SNF patients.

## Discussion

### Summary of findings and clinical implications

These 2 cases illustrate the potential of ForeSight-HF to provide bidirectional clinical decision support across contrasting Forrester hemodynamic quadrants, encompassing both therapy intensification and de-escalation. ForeSight-HF addresses several gaps in current SNF HF care. First, it provides risk stratification that enables appropriate triage in settings without daily specialist oversight. The risk scores demonstrated the system's potential to discriminate between low-acuity optimization opportunities and high-acuity decompensations requiring urgent intervention. Second, this bidirectional guidance addresses the full spectrum of management errors in SNF care. Although undertreatment of HF is well-documented,^9^ overtreatment with excessive diuresis represents a significant iatrogenic risk in frail older patients.

### Comparison with prior work

To our knowledge, ForeSight-HF represents the first AI-CDSS designed specifically for postacute care rather than acute hospital settings. Its large language model architecture enables processing of unstructured clinical documentation without requiring structured data inputs, a practical advantage in SNF settings where structured electronic health record (EHR) data are often unavailable.

### Study limitations

First, both cases were purposively selected to illustrate system performance across contrasting Forrester quadrants, introducing potential selection bias. Second, ForeSight-HF is not currently integrated with EHR systems; reliance on manual data input may affect utility and introduces potential for transcription errors. Future integration with EHR systems to enable automated, seamless data ingestion represents an important next step to improve accuracy and scalability. Third, only 2 of the 4 Forrester quadrants are represented, with 1 case per quadrant. System performance across larger, more diverse SNF populations remains to be established.

## Conclusions

This 2-case report demonstrates that ForeSight-HF can provide bidirectional clinical decision support based on Forrester quadrants—from diuretic intensification for congestion (Warm/Wet) to therapy de-escalation in overdiuresed patients (Cold/Dry). The system is not intended to replace clinical judgment, but rather to facilitate early diagnosis, GDMT optimization, and timely escalation when needed. Prospective validation studies are warranted to address critical unmet needs in postacute HF care.

## Funding Support and Author Disclosures

This work received no external grant funding. Platform infrastructure was provided by ProviderLoop, New York, New York. Dr Bander is the founder and holds an ownership interest in ProviderLoop, the platform on which ForeSight-HF was developed. All other authors have reported that they have no relationships relevant to the contents of this paper to disclose.
